# Knowledge, attitudes, practices and information demand in relation to haze in China: a cross-sectional study

**DOI:** 10.1186/s12889-019-7772-3

**Published:** 2019-10-28

**Authors:** Minghui Zhao, Meiling Zhang, Jie Ying, Shouqi Wang, Ying Shi, Huanhuan Li, Yuan Li, Zhuangjie Xing, Jiao Sun

**Affiliations:** 1 Department of Endocrinology, The Second Hospital of Jilin University, Changchun, 130022 China; 2 College of Humanities & Sciences of Northeast Normal University, Changchun, 130117 China; 3 Hohhot Vocational College, Hohhot, 010051 China; 4 Department of operating room, The Second Hospital of Soochow University, Suzhou, 215000 China; 5 Hubei University of Medicine, Shiyan, 442000 China; 6 School of Nursing, Jilin University, Changchun, 130021 China

**Keywords:** Haze, Knowledge, Attitudes, Practices, Information demand

## Abstract

**Background:**

Information on the effects of haze on health and corresponding knowledge, attitudes, and practices (KAP) will improve self-care ability against the ill effects of haze pollution and make environmental health policies more targeted and effective. In this study, we aim to determine the KAP and information demand in the context of haze and its effects on health.

**Methods:**

A cross-sectional survey was conducted in Changchun, China, between October and November 2016. A purposive sample comprising four groups (college students, occupational population, traffic police, and elderly population) were investigated. Personal information and questions pertaining to KAP and information demand on haze pollution and health were collected through questionnaire.

**Results:**

The questionnaire was completed by 888 residents. The awareness rate was 57.7% and varied significantly with education, exercise, and population group (*p* < 0.05). Among the four groups, college students scored the lowest in attitudes and practices, whereas traffic police scored the highest. Concerning the source of information demand, the elderly and traffic police preferred traditional information access (i.e., television and newspaper), whereas college students and the occupational population preferred new social media (i.e., mobile newspaper and social media sites). Regarding the most trusted department that releases information, all residents relied on the haze information released by the environmental protection department and meteorological department. The latest air quality was the most desired information of the residents.

**Conclusions:**

Changchun residents have a relatively high knowledge awareness rate. The elderly and less-educated residents are the targetable population for haze education. Education should be disseminated in such manner as to meet the needs of different people.

## Background

Haze pollution is an increasingly severe public health problem worldwide, especially in China, where environmental deterioration has accompanied rapid economic growth. The fundamental cause of haze is the high concentration of fine particles (PM_2.5_) or aerosol pollution mainly caused by coal combustion, vehicle exhaust, and industrial emissions. Among all the contributing factors, coal-dominated energy structures are the worst offenders, accounting for nearly 70% of the primary energy consumption in China [1]. In 2010, air pollution ranked ninth among the global risk factors that cause disease burden in the entire world and fourth in East Asia [2]. The WHO estimated that three million deaths globally in 2012 were caused by air pollution, and China alone contributed 1.03 million [3].

In China, serious pollution events often occur during winter in the north. An example is Changchun (125°E, 43°N), which is the capital city of Jilin Province and an important industrial base. Given that the city has a temperate continental semihumid season, coal heating is required for approximately half a year, and the large amounts of black carbon released from coal heating further aggravate the formation of haze pollution [4]. In 2015, the China Environmental Aspect Bulletin reported that, among the 74 cities monitored by the new standards, the Changchun air quality composite index ranked 54th. Its average annual PM_2.5_ concentration of 66 μg/m^3^ was three times that of Haikou who ranked first and exceeded the national level II standard 0.89 times [5]^.^ In 2016, the air quality situation released by the Changchun Environmental Protection Bureau showed that the average annual value of PM_2.5_ in Changchun was 46 μg/m^3^, which exceeded the national level II standard 0.31 times [6]. Although the air quality slightly improved at present, haze pollution in Changchun remains a continuing public health concern.

In response to the current severe haze pollution situation, the State Council in China issued its Action Plan on the Prevention and Control of Air Pollution (the “Action Plan”) [7], which stated that the PM_2.5_ concentrations in Beijing, Nanjing, and Guangzhou should be reduced by 25, 20, and 15%, respectively, between 2012 and 2017. For the efficient implementation of the policy, understanding public perceptions is essential as they are important factors in the successful implementation of environmental policies [8, 9]. If the public view of risk is not included in the policy, then the execution of environmental policies will face serious challenges [10]. Survey data on the knowledge, attitudes, and practices (KAP) are essential in the planning, implementation, and evaluation of a program and identification of knowledge gaps, cultural beliefs, or behavioral patterns that may facilitate or impede the success of that program [11]. A study of KAP is critical to the development and implementation of haze policy.

A handful of previous studies on KAP related to haze pollution focused on a specific single group. A knowledge survey that involved 2140 high-school students in Tehran, Iran, revealed that their knowledge about the composition of clean air is incorrect. For example, approximately 50% of the students identified ozone and approximately 40% of the students identified carbon monoxide as components of clean air; in fact these gases are the main air pollutants [12]. A cross-sectional survey in Peninsular Malaysia pointed that general attitudes towards haze are highly negative among its population. The marked public discomfort with the phenomenon is starkly revealed by the 42% of respondents who reported that they had considered leaving the country because of the effects of haze [13]. In a study in Nepal [14], 88.0% of the traffic police respondents have below-average level of practice regarding the prevention of respiratory problems due to haze, although 75.9% of them have at least an average level of knowledge.

In recent years, with the rapid development of science and technology, health knowledge is transmitted in an increasing number of ways. The traditional means of information dissemination interleave with the modern ones. Qian et al. [15] revealed that the most commonly reported sources of haze information of 57.42% of residents are television and internet resources, whereas 22.88% of residents obtain haze information from books and newspapers and 19.70% from expert lectures and friends. Yang et al. [16] showed that mainstream media (such as television and newspaper), accounting for 67% of all media sources, are the main sources of haze information. In addition, a survey in Beijing found that television is the most popular means by which 70.8% of residents obtain information about haze prevention and control [17]. The results of the preceding studies revealed the most preferred sources of haze information only but not the specific needs of the residents. Therefore, the demand of residents for information on haze pollution must be explored in depth.

To date, the KAP of diverse groups of people regarding haze pollution is currently limited. Moreover, data is lacking on the demand of information regarding haze pollution. This study was directed such that this issue is explored particularly for the understanding and identification of factors associated with KAP and information demand on haze pollution in Changchun. The findings of this study can help healthcare workers identify and rectify incorrect knowledge and behaviors of residents and understand their needs. Given that different groups have different self-care defects, targeted interventions and health education is essential to improving self-care and reducing haze risk. This information can also be vital to local governments with respect to the development of policies and effective measures for reducing the impact of haze in the future.

## Methods

### Study design and sample

A cross-sectional survey was conducted and aimed at investigating the KAP and information demand of the residents in Changchun, China in relation to haze pollution. Four different groups (college students, occupational population, traffic police, and elderly population) were selected as survey subjects. A university, state-owned enterprise, traffic police division, and senior center were selected for the investigation. Four survey sites in Chaoyang District were randomly selected from nine districts in the urban area of Changchun. Meanwhile, 996 respondents were selected through purposive sampling. We ensured that the sample included people who are regularly outdoors, those who are not, and those in good health, and those in poor health for comparison. Additional inclusion criteria were as follows: (1) willing to participate in the study, (2) 18 years old or older, (3) registered permanent Changchun resident (living at their present residence for more than 1 year in Changchun before this survey), and (4) mentally fit to cooperate with the investigation. Those who were qualified briefly informed about our study, and their consent were obtained. The data were collected between October and November 2016.

### Instrumentation

For the cross-section survey, we conducted a face-to-face interview to assess the residents’ KAP and information demand with regard to haze and health. The surveys were extensively pretested prior to study through the implementation for the validation of the questionnaires. The validity of the questionnaire was established through content and expert validity. Cronbach’s alpha values for testing the internal consistency of KAP outcomes were between 0.778 and 0.907. After extensive literature review, A 59-item questionnaire designed by nursing experts was used. This questionnaire was based on the questionnaire of Qian et al. [15] and focused primarily on the following aspects:

### Part 1: social demographics

Eight items in Part 1 gathered general information, such as age, gender, and educational level.

### Part 2: knowledge related to basic concepts, hazards, and protective measures

A total of 29 items in Part 2 inquired about the residents’ knowledge of the basic concepts and hazards of and protective measures against haze pollution. A correct answer was scored 1, and an incorrect answer was scored 0. The scores varied from 0 to 29 points, and a high score indicated high knowledge. The scores were classified into two levels (high knowledge level for 17.4–29 points and low knowledge level for 0–17.3 points) [15, 18]. The standard of the high knowledge level ranged from 60 to 100%, while the standard of the low knowledge level was 60% or less. The knowledge awareness rate was defined in this study as the percentage of respondents who were classified into high knowledge level.

### Part 3: attitudes regarding the haze information attention and protection

Part 3 was composed of six items on a five-point Likert scale that examined the attitudes of the residents towards information attention and protection against haze in a range of 6 to 30. A high score indicated a positive attitude.

### Part 4: practices of protective behaviors against haze pollution

Part 4 was composed of 12 items on a five-point Likert scale that examined the self-protective health and public health behaviors in a range of 12–60. A high score indicated improved practice. The scores were classified into two levels (good practice for 48.1–60 points and moderate practice for 12–48 points). The standard of good practice levels ranged from 81 to 100%. The standard of moderate practice levels was 80% or less.

### Part 5: information demand regarding haze pollution

Part 5 was composed of four questions that inquired about the most trusted department that releases information, desired information, and source of haze information.

### Quality control

Eight authors with two members each conducted survey in four locations. After the survey, two data collectors (Y.S and J.Y) reviewed and numbered the questionnaires to facilitate data entry and statistical analysis.

### Sample size and data analysis

The formula for estimating the sample size as follows: *n* = [μ_α_^*2*^ *p* (*1-p*)/δ^*2*^] × (1 + 20%), α = 0 .05, μ_α_ = 1.96, *p* = 0.6459, δ = 0.15*p*, *n* ≈ 120. The study needed four groups and a total of 480 residents.

The completed questionnaires were imported into a computer by using EpiData 3.1, and double data entry was used for error reduction. The Statistical Package for the Social Sciences (SPSS) version 23.0 was used for data analysis. The demographic data and KAP of the residents towards haze pollution were analyzed using frequency percentage. Normal distributions of continuous variables were tested using the Kolmogorov–Smirnov test. The data were skewed distribution, and therefore, median ± quartile were used. Chi-square tests were applied for the demographic factors, including age, gender, and educational level. Multiple unconditional logistic regression was used for the assessment of the associations between knowledge outcome and demographic factors, and the adjusted odds ratio (OR) and 95% confidence interval (CI) were presented. The alpha level was set at 0.05 to determine statistical significance.

### Ethics statement

Ethics approval for this participant contact study was obtained from the Research Ethics Committee of the Nursing School of Jilin University. Written consent was not required, and verbal agreement to participate in the survey was considered as consent. The participants were informed before commencement that they can withdraw at any stage while being surveyed and that such withdrawal will render their entire participation void. They were also informed of the anonymity of the data collection.

## Results

The general demographic characteristics are summarized in Table 1. The study was conducted on a total of 996 subjects (college students, 298; occupational population, 350; traffic police, 188; elderly population, 160). A total of 888 questionnaires were completed (college students, 267; occupational population, 320; traffic police, 161; elderly population, 140), and the response rate was 89.16%. The age of the participants ranged from 18 years to 91 years with a mean of 39.6 ± 1.9 years. Of the 888 subjects, 28.4% were males and 71.6% were females. More than 80% of the residents obtained a college education. College students with a bachelor’s degree accounted for 97%, whereas the corresponding proportion of the elderly accounted for 66.4%. The results showed that just plenty of residents never do any exercise: 11.2% for the college students, 6.6% for the occupational population, 18.0% for the traffic police, and 2.1% for the elderly population. Only a few residents (27.7%) exercise ≥3 times in a week. A total of 77.4% of the study participants perform outdoor activities; others (22.6%) exercised indoors. Overall, most participants (65.5%) were healthy.

**Table 1:** General demographic information of the four groups, n (%)

Demographic factors			College students	Occupational population	Traffic police	Elderly population	Total
Number of surveys			267	320	161	140	888
Age, mean ± SD			20.4 ± 2.2	43.5 ± 10.2	41.4 ± 8.9	65.6 ± 6.8	39.6 ± 1.9
Sex		Male	17 (6.4)	57 (17.8)	140 (87.0)	38 (27.1)	252 (28.4)
		Female	250 (93.6)	263 (82.2)	21 (13.0)	102 (72.9)	636 (71.6)
Level of education		<High school	8 (3.0)	43 (13.4)	19 (11.8)	47 (33.6)	117 (13.2)
		≥College	259 (97.0)	277 (86.6)	142 (88.2)	93 (66.4)	771 (86.8)
Exercise		No	30 (11.2)	21 (6.6)	29 (18.0)	3 (2.1)	83 (9.3)
		Yes	237 (88.8)	299 (93.4)	132 (82.0)	137 (97.9)	807 (90.7)
Exercise frequency		< 3 times a week	211 (89.0)	198 (66.2)	104 (78.8)	69 (50.4)	582 (72.3)
		≥3 times a week	26 (11.0)	101 (33.8)	28 (21.2)	68 (49.6)	223 (27.7)
Exercise location		Indoors	73 (30.8)	63 (21.1)	26 (19.7)	20 (14.6)	182 (22.6)
		Outdoors	164 (69.2)	236 (78.9)	106 (80.3)	117 (85.4)	623 (77.4)
Illness		No	224 (83.9)	202 (63.1)	96 (59.6)	60 (42.9)	582 (65.5)
		Yes	43 (16.1)	118 (36.9)	65 (40.4)	80 (57.1)	306 (34.5)
Attitude	Q1	Strongly disagree/disagree	80 (30.0)	88 (27.5)	50 (31.0)	40 (28.5)	258 (29.1)
		Neither	147 (55.1)	172 (53.8)	86 (53.4)	74 (52.9)	479 (53.9)
		Strongly agree/agree	40 (14.9)	60 (18.8)	25 (15.5)	26 (18.6)	151 (17.1)
	Q2	Strongly disagree/disagree	44 (16.4)	11 (3.5)	10 (6.2)	5 (3.5)	70(7.9)
		Neither	142 (53.2)	89 (27.8)	36 (22.4)	45 (32.1)	312 (35.1)
		Strongly agree/agree	81 (30.3)	220 (68.8)	115 (71.5)	90 (64.3)	506 (57.0)
	Q3	Strongly disagree/disagree	35 (13.1)	8 (2.5)	2 (1.2)	1 (0.7)	46 (5.2)
		Neither	143 (53.6)	111 (34.7)	42 (26.1)	47 (33.6)	343 (38.6)
		Strongly agree/agree	89 (33.3)	201 (62.8)	117 (72.7)	92 (65.7)	499 (56.2)
	Q4	Strongly disagree/disagree	44 (16.5)	14 (4.4)	5 (3.1)	11 (7.8)	74 (8.3)
		Neither	114 (42.7)	110 (34.4)	43 (26.7)	47 (33.6)	314 (35.4)
		Strongly agree/agree	109 (40.8)	196 (61.2)	113 (70.2)	82 (58.6)	500 (56.3)
	Q5	Strongly disagree/disagree	11 (4.1)	8 (2.5)	3 (1.9)	4 (2.8)	26 (2.9)
		Neither	71 (26.6)	29 (9.1)	29 (18.0)	19 (13.6)	148 (16.7)
		Strongly agree/agree	185 (69.3)	283 (88.4)	129 (80.1)	117 (83.6)	714 (80.4)
	Q6	Strongly disagree/disagree	20 (7.5)	5 (1.5)	2 (1.2)	6 (4.3)	33 (3.7)
		Neither	108 (40.4)	93 (29.1)	40 (24.8)	47 (33.6)	288 (32.4)
		Strongly agree/agree	139 (52.1)	222 (69.4)	119 (73.9)	87 (62.1)	567 (63.8)
Practice levels		Good practice	77 (28.8)	147 (45.9)	69 (42.9)	59 (42.1)	352 (39.6)
		Moderate practice	190 (71.2)	173 (54.1)	92 (57.1)	81 (57.9)	536 (60.4)

Attitude Q: Attitude Question

1. Are you satisfied with the air quality in Changchun?

2. Will you know the latest haze situation issued by the authorities timely and accurately?

3. Have you paid attention to the haze weather?

4. Will you take protective measures initiatively?

5. Are you willing to reduce the impact of haze pollution through your own efforts?

6. Do you think every citizen is responsible for the haze pollution?

### Attitudes

Only 17.1% of the residents expressed satisfaction with the air quality in Changchun. More than half of the residents, especially the traffic police, updated themselves with the latest haze situation and paid attention to the haze weather. Most residents practiced protective measures initiatively when exposed to haze pollution, and 80% of the residents were willing to reduce the impact of haze pollution through their own efforts. Approximately 63.8% of the residents agreed that every citizen is responsible for the haze.

### Practices

The percentage of the protection practices of the residents against haze pollution is summarized in Table 1. Among the 888 residents, 39.6% of them exhibited good practice and nearly two-thirds exhibited moderate practice. The practice scores in the group of college students were significantly different from those of the groups (*χ*^2^ = 19.386 *p* < 0.05).

### KAP differences

Results (Table 2) of the nonparametric test showed that the KAP scores in the four groups differed significantly (*p* < 0.001). The students, occupational population, and traffic police scored high levels of knowledge, and no significant difference was observed among the levels (*p* > 0.05). Nevertheless the knowledge score of the elderly was low, and the difference was significant compared with others (*p* < 0.001). The attitude scores of the occupational population, traffic police, and elderly did not differ significantly (*p* > 0.05). However, the attitude score of the students was lower than and differed significantly from that of the other three groups (*p* < 0.05). The occupational population, traffic police, and elderly obtained good practice scores, which did not differ significantly (*p* > 0.05). Whereas the students obtained a moderate practice score, which differed significantly from that of the other three groups (*p* < 0.05). Although the differences of college students’ attitude and practice scores compared with the other three are small, they are still meaningful.

**Table 2:** Differences between the KAP scores of the four groups

	Knowledge			Attitudes			Practices		
	*M(P*_*25*_ *, P*_*75*_ *)*	*Η*	*p-value*	*M(P*_*25*_ *, P*_*75*_ *)*	*Η*	*p-value*	*M(P*_*25*_ *, P*_*75*_ *)*	*Η*	*p-value*
College students	19.00/(17.00,21.00)**	30.606	<0.001	20.00/(18.00,22.00)	105.52	<0.001	46.00/(40.00,50.00)	41.28	<0.001
Occupational population	18.00/(16.00,21.00)**			23.00/(20.00,25.00)*			48.00/(45.00,54.00)*		
Traffic police	19.00/(15.00,23.00)**			23.00/(20.00,26.50)*			48.00/(46.00,57.00)*		
Elderly population	16.00/(13.00,19.75)			22.00/(20.00,24.00)*			48.00/(44.00,52.00)*		

The values of multiple comparisons were based on the Dunn–Bonferroni post-hoc test: *p < 0.05 versus college students;

**p < 0.001 versus elderly population; M:Median

### Awareness rate

The awareness rate to haze and its related knowledge was 57.7% (512/888). The awareness rates of the four groups differed significantly. The students demonstrated the highest level of awareness, which accounted for 64.8% (*p* < 0.001). Although awareness declined with increasing age, but there was no statistical significance. Moreover, awareness increased significantly with the improvement of education level (*p* < 0.001) and differed significantly by exercise (*p* < 0.05). However, no significant difference was found in the awareness rate between men and women, age groups, and other aspects (*p* > 0.05) (Table 3).

**Table 3:** Comparisons of the residents’ demographic in relation to awareness rate

Characteristics		n (%)	Awareness rate n (%)	*χ* ^*2*^	*p-value*
Population group	College students	267 (30.1)	173 (64.8)	25.347	<0.001
	Occupational population	320 (36.0)	190 (59.4)		
	Traffic police	161 (18.1)	94 (58.4)		
	Elderly population	140 (15.8)	55 (39.3)		
Age, (years)	<20	78 (8.8)	47 (60.3)	4.131	0.248
	20–40	371 (41.8)	221 (59.6)		
	40–60	299 (33.7)	173 (57.9)		
	≥60	140 (15.8)	70 (50.0)		
Sex	Male	252 (28.4)	134 (53.2)	2.751	0.097
	Female	636 (71.6)	377 (59.3)		
Level of education	≤High school	117 (13.2)	48 (41.0)	15.052	<0.001
	≥College	771 (86.8)	463 (60.1)		
Exercise	No	83 (9.3)	59 (71.1)	6.87	0.010
	Yes	805 (90.7)	452 (56.1)		
Exercise frequency	<3 times a week	582 (72.3)	341 (58.6)	0.289	0.591
	≥3 times a week	223 (27.7)	126 (56.5)		
Exercise location	Indoors	182 (22.6)	106 (58.2)	0.005	0.943
	Outdoors	623 (77.4)	361 (57.9)		
Illness	No	582 (65.5)	345 (59.3)	2.077	0.154
	Yes	306 (34.5)	166 (54.2)		

### Variables associated with awareness rate

Multiple unconditional logistic regression analysis was performed for the identification of demographic factors associated with awareness rate; data are shown in Table 4. Only one variable significantly influenced the knowledge awareness rate: population group. The knowledge awareness rate of the students was established as the baseline, and the knowledge awareness rates of other groups were lower.

**Table 4:** Unconditional multivariate logistic regression analysis for the association between sociodemographic and haze pollution awareness rate

Characteristics	β	OR	95%CI ^a^	*p-value*
Population group				
College students (reference)		1.00	0.00–0.00	
Occupational population	−6.431	0.00	0.00–0.01	<0.01
Traffic police	−4.477	0.01	0.00–0.08	<0.01
Elderly population	−6.97	0.00	0.00–0.01	<0.01

^a^*CI* confidence interval, *OR* odds ratio. Note: assigned “the score of knowledge ≥17.4” to 1; others to 0

### Source of information

**Fig. 1:**
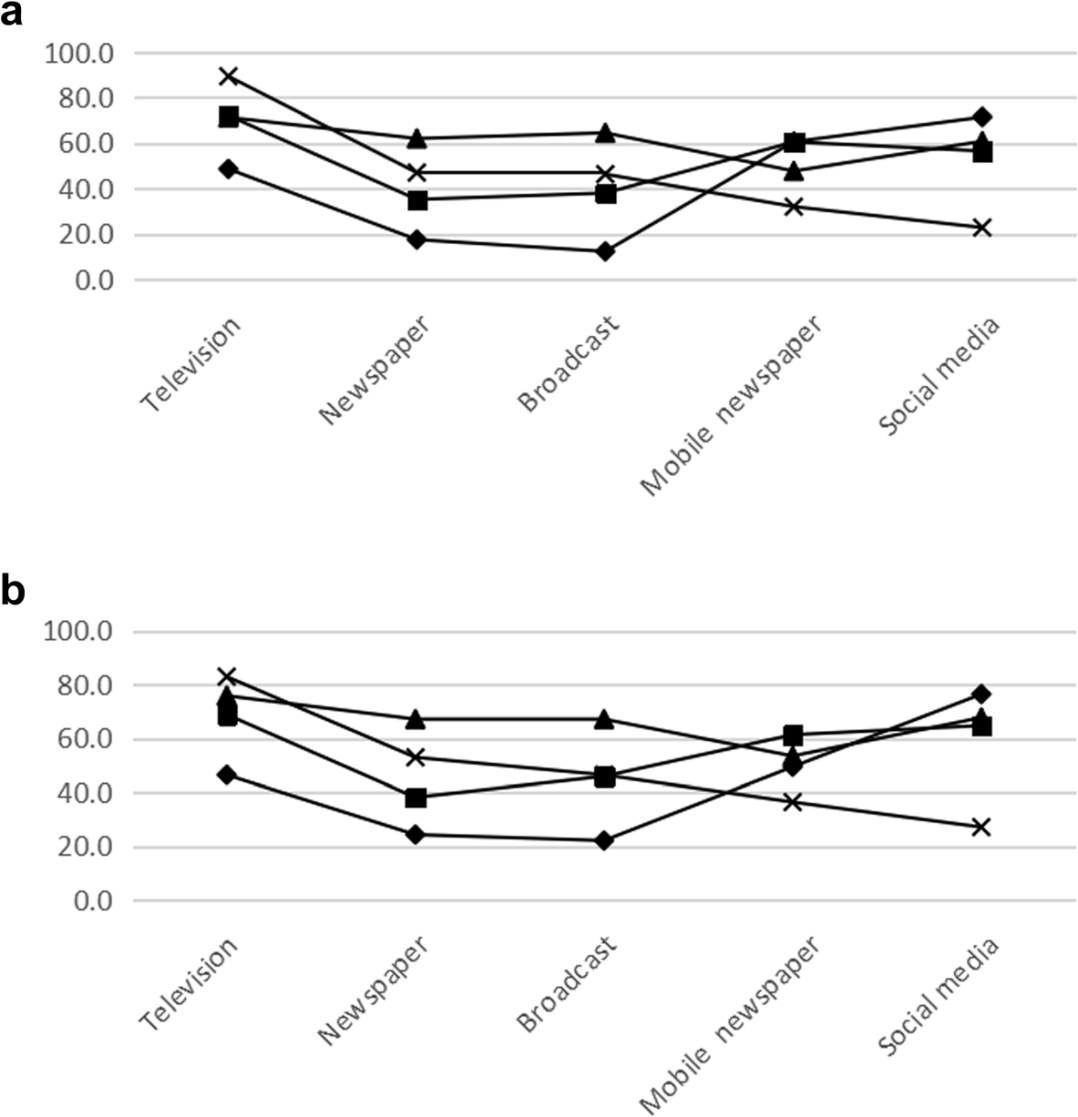
**a** Source of information. **b** Expected source of information. ♦ College students ■ Occupational population. ▲ Traffic police **×** Elderly population

For the source of haze information, the elderly and traffic police preferred traditional information access, such as television (89.3 and 71.4%, respectively), newspaper (47.1 and 62.1%, respectively), and broadcast (46.4 and 64.6%, respectively). The students and occupational population preferred new social media, such as mobile newspaper (60.7 and 60.6%, respectively) and social media sites (71.5 and 56.6%, respectively). Television was the most popular source. The results of the ideal source of information were similar to aforementioned results (Fig. 1).

### Demand of information

**Fig. 2:**
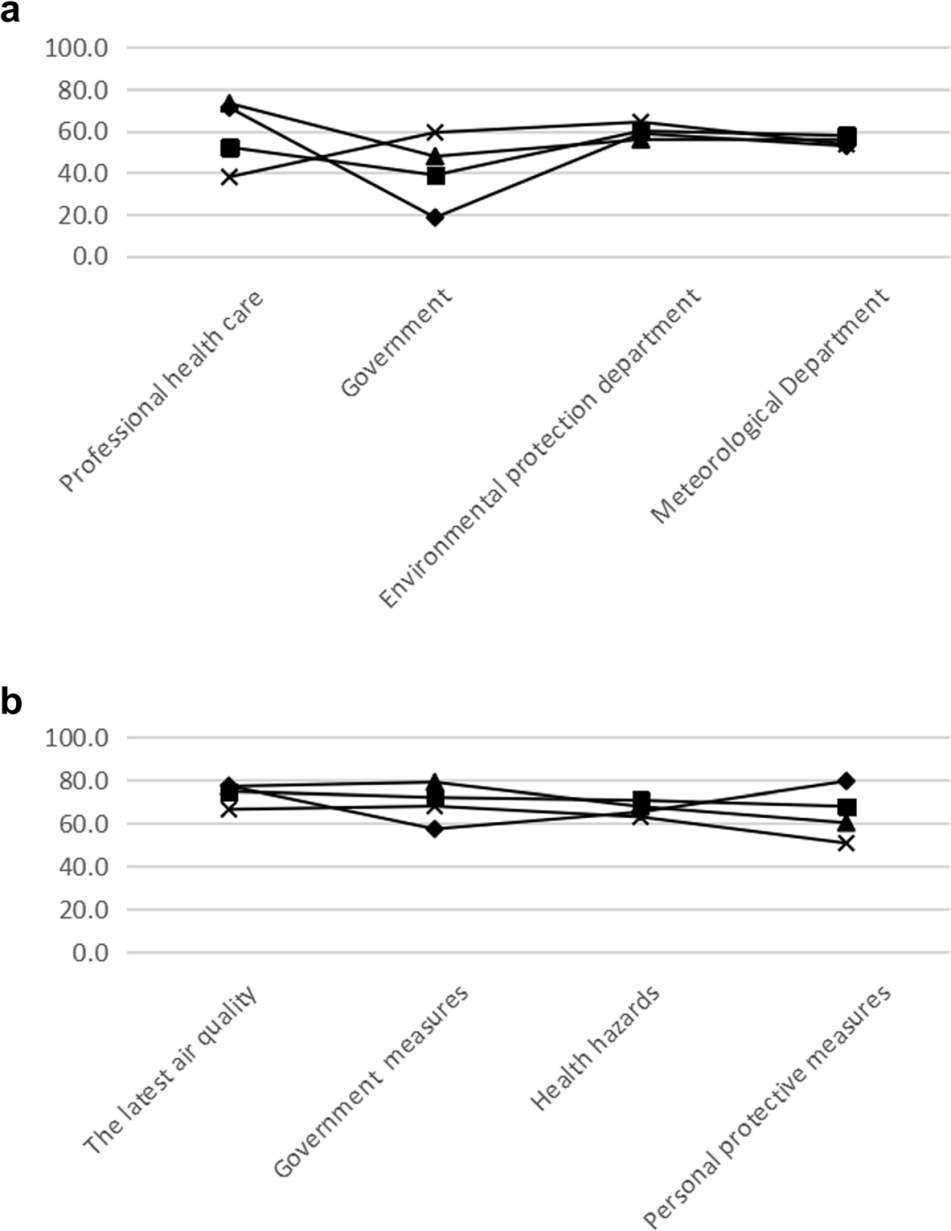
**a** Trusted department that released information. **b** Desired information. ♦ College students ■ Occupational population. ▲Traffic police, **×** Elderly population

All residents relied on the haze information released by the environmental protection department (college students 58.8%, occupational population 60.0%, traffic police 55.9%, and the elderly 64.3%) and meteorological department (college students 52.8%, occupational population 57.8%, traffic police 56.5%, and the elderly 53.6%). The trust levels of residents on the environmental protection department and the meteorological department were basically flat. The college students (71.2%) and traffic police (73.35%) were more likely to trust healthcare professionals than the occupational population (52.2%) and elderly (37.9%). However, the residents’ reliance on the government was generally low. The attention rates of the four groups to the latest air quality (college students 77.2%, occupational population 74.7%, traffic police 77.0%, and the elderly 66.4%) and health hazards (college students 65.2%, occupational population 70.6%, traffic police 67.7%, and the elderly 62.9%) were basically the same. College students paid less attention to government measures (57.3%) but exhibited high concern for personal protection measures (79.4%) (Fig. 2).

## Discussion

This study utilized a questionnaire survey to compare residents’ KAP and information demand on haze in Changchun. In contrast to previous study conducted in China [11, 15], our study was based on four different groups of people. We analyze the factors that affect residents’ awareness and attempted to improve residents’ knowledge of haze protection, thereby promoting the formation of their attitude and behavior. And the finding of information demand are important factors in the successful implementation of environmental policies in north China.

Previous studies have shown a low awareness rate (29.8%) to PM_2.5_ in Harbin residents [19]. However, in the current survey, Changchun residents showed a high awareness level about haze pollution. This outcome was primarily due to the hazardous levels of haze that Changchun residents suffered in 2013 [20]. Such experience encouraged the local residents to obtain a better understanding of haze pollution. Notably, the differences among the levels of knowledge of haze affected the awareness rates. Among the four groups, college students obtained the highest awareness rate and knowledge score in the present study. Conversely, two studies found that the awareness rate and knowledge of college students on haze are not ideal [21, 22]. This difference in results can be attributed to the location and weather condition in Changchun. Coal burning is longer, and consequently people are exposed to haze longer than the subjects in the two aforementioned research sites. Thus, students in Changchun have more knowledge about haze pollution. However, the awareness rate and knowledge score of the elderly were significantly lower than those of the other three groups. This outcome was possibly due to their traditional manner of accessing knowledge related to haze; hence, they cannot obtain the latest haze information timely and accurately. Therefore, the elderly should maximize the use of all available resources to gain knowledge, try new ways to get information, communicate more with others, and attend community or hospital health talks.

In this study, only 17.1% of the residents expressed satisfaction with the air quality in Changchun. However, 80% of the residents were willing to reduce the impact of haze pollution through their own efforts, and 63.8% of the residents agreed that every citizen is responsible for the haze. These findings were coincident with those of previous studies [15, 18]. Therefore, most residents have high awareness and sense of obligation to engage and participate in managing the haze pollution. Moreover, participation in air quality monitoring helps residents improve their understanding of air pollution and develop responses to it [23]. Although the city rarely experiences an extreme haze weather, all its residents are highly knowledgeable about health hazards. Thus, more than half of the residents obtained the latest haze situation and paid attention to the haze weather; these results were similar to those of Cao et al. [24] and Qian et al. [25]. A total of 56.3% of the residents practiced protective measures initiatively when exposed to haze pollution. The increasing number of people who have begun to focus on and prevent the negative impacts of haze pollution is promising.

The results of this study showed that 39.6% of the residents obtained a good practice score and nearly two-thirds obtained a moderate practice score. Among the four groups, the practice scores of college students significantly differed from those of the other groups (*χ*^2^ = 19.386 *p* < 0.05), although they obtained the highest awareness rate and knowledge score among the four groups. An apparent disconnect between knowledge and practice was observed. Kanyiva et al. came to the same conclusion; they found that the residents recognize that they need to open the windows and doors when cooking using a kerosene or charcoal stove to vent the emissions but do not practice it [26]. Similarly, Niu et al. [27] showed that policy makers in Northwest China identified that children are uniquely vulnerable to air pollution, yet few of them know the policies to protect the children. In summary, haze pollution education is important, not only to provide residents with the basic knowledge of haze but also to inform them of the appropriate actions to take to protect themselves. Moreover, college students should develop the habit of paying attention to weather forecasts and take measures, such as wearing face masks in haze weather.

Pretto et al. [13] and Wang et al. [18] reported a high level of awareness and concern over air pollution encourages people to engage into protective actions against it. A positive correlation exists between the two. Similar to previous research results, the findings of the current study showed that traffic police had the highest scores in attitudes and practices possibly because they are often outdoors and often exposed to haze pollution and thus have a strong sense of self-protection.

The data of the current study were identical with previous literature that television is the most popular way to access haze information and the Internet is replacing traditional newspapers and other means as the most common ways to obtain knowledge about haze pollution [15, 16, 28], especially in college students and occupational population. These two groups were more inclined to use more visual, auditory and intuitive ways than the other groups in order to obtain information, such as mobile newspaper and social media sites. However, the elderly and traffic police preferred traditional information access, such as television, newspaper, and broadcast. This illustrated the changes in the way information is disseminated with the development of society. To the best of our knowledge, only two studies have assessed the specific information demand of residents towards haze pollution [17, 28]. In contrast with their results, this study found that confidence in the government was generally low and the latest air quality was the most desired information of the residents. The needs varied from person to person. All these findings suggested that the government should meet the different demands of the residents.

This study found that the residents with a high educational level exhibited a high awareness level in the single variable analysis; this finding was consistent with the results of prior studies regarding the association of knowledge awareness rate with education [18, 29–31]. Meanwhile, a study has shown that education level had no significant effect on haze awareness rate [13], which is consistent with the results of our multivariable analysis. But a study from the United States indicated that individuals with low educational attainment are more perceptive and responsive to air quality than those with high education attainment [32]. Thus, further studies are needed to clarify these differences.

Controversy exists with regards to the association of knowledge awareness rate with age. This study indicated that the awareness rates to haze pollution between the youth and the elderly did not differ significantly; this finding was similar that of previous studies that have not found a significant association between age and perceptions of air quality [33, 34]. Other studies observed that the youth have poor awareness rate compared with the elderly [17, 30]. However, most of these studies have not given a clear explanation for these differences. Further research needs to investigate the interaction between age and awareness rate to haze pollution.

Although exercise is known to be highly beneficial to health [35–38], engaging in it in a polluted environment may increase population-wide health risks [39, 40]. Pretto et al. [13] reported that people who regularly practice outdoor sports are more knowledgeable about haze than people who exercise indoors. However, this study found that people who did not exercise were more aware of the haze than people who exercised. After inclusion of multivariate analysis exercise was no longer meaningful. The relationship between exercise and awareness rate is controversial possibly due to the lack of uniform assessment criteria for awareness rate and the different control of exercise frequency and mode. Thus, further research is needed to help understand this factor shaping people’s awareness.

Considering the residents’ occupations, a previous study has shown that the retired and the manual laborers exhibit a lower degree of concern and awareness rate than those groups as the students, cadre, and technicians [15]. This finding was consistent with the findings of the current study. Among the four groups, the awareness rates of the college students and occupational population were higher than those of the traffic police and elderly. This outcome was possibly due to the influencing factors of education level, economical status, and social status.

The influence of gender on awareness rate had no statistical significance based on the results of this study. This was contrary to previous researchs [31, 41, 42], which found that female had a higher level of risk perception of air pollution than male. The reason for the difference may be the high proportion of female in the sample. Thus, further research needs to explore the interplay between gender and awareness rate to haze pollution.

### Limitations of this study

The study employed purposive sampling of four groups of people, and thus the sample was not representative of all the residents in Changchun. People with bachelor’s degree or higher educational level in the sample accounted for 86.8%. The findings may, therefore, have limited relevance for residents with low educational levels. In addition, representatives of outdoor workers should not only be limited to traffic police; sanitation workers, taxi drivers, etc., may have different KAP about haze pollution. Moreover, the questionnaires used in this survey did not include open-ended questions. The question style failed to reveal new problems and deepen the understanding of the haze situation.

Unfortunately, economic status was not addressed in the study, and differences possibly existed between the high-income and low-income groups. However, the student groups included in this study did not have a source of income; hence, economic status cannot be included as an aside in this article. An in-depth consideration of the effect of economic status on the awareness rate to haze pollution is a subject for further research.

## Conclusions

A total of 57.7% of the residents of Changchun were aware of haze pollution. However, the KAP scores among the four groups were statistically significant and relatively low in some groups, such as the less educated and elderly. These results emphasized the importance of improving the awareness of residents toward haze pollution through public education and environmental protection campaigns.

On the source of haze pollution, the elderly and traffic police preferred traditional information access, whereas the students and occupational population preferred new social media. Therefore, health education for the prevention and control of haze pollution should be provided in different ways to diverse populations. The reliance of the residents on the government was generally low. This finding suggested that the government should allocate more resources towards educating citizens of the haze pollution problem, providing them with appropriate practices to help lessen the detrimental effects, and encouraging people to actively participate in and monitor haze pollution control to achieve continuous improvement of air quality.

## Data Availability

The datasets used and analysed during the current study are available from the corresponding author on reasonable request.

## Abbreviation

KAP: knowledge, attitudes, and practices
